# Taxonomic difference in marine bloom-forming phytoplanktonic species affects the dynamics of both bloom-responding prokaryotes and prokaryotic viruses

**DOI:** 10.1128/msystems.00949-23

**Published:** 2024-03-05

**Authors:** Hiroaki Takebe, Kento Tominaga, Tatsuhiro Isozaki, Tetsuhiro Watanabe, Keigo Yamamoto, Ryoma Kamikawa, Takashi Yoshida

**Affiliations:** 1 Graduate School of Agriculture, Kyoto University, Kyoto, Japan; 2 Graduate School of Frontier Sciences, The University of Tokyo, Tokyo, Japan; 3 Research Institute of Environment, Agriculture and Fisheries, Osaka Prefecture, Osaka, Japan; UiT - The Arctic University of Norway, Tromsø, Norway

**Keywords:** phytoplankton bloom, prokaryotes, prokaryotic viruses, microcosm, *Heterosigma akashiwo*, *Chaetoceros* sp.

## Abstract

The production of dissolved organic matter during phytoplankton blooms and consumption by heterotrophic prokaryotes promote marine carbon biogeochemical cycling. Although prokaryotic viruses presumably affect this process, their dynamics during blooms are not fully understood. Here, we investigated the effects of taxonomic difference in bloom-forming phytoplankton on prokaryotes and their viruses. We analyzed the dynamics of coastal prokaryotic communities and viruses under the addition of dissolved intracellular fractions from taxonomically distinct phytoplankton, the diatom *Chaetoceros* sp. (CIF) and the raphidophycean alga *Heterosigma akashiwo* (HIF), using microcosm experiments. Ribosomal RNA gene amplicon and viral metagenomic analyses revealed that particular prokaryotes and prokaryotic viruses specifically increased in either CIF or HIF, indicating that taxonomic difference in bloom-forming phytoplankton promotes distinct dynamics of not only the prokaryotic community but also prokaryotic viruses. Furthermore, combining our microcosm experiments with publicly available environmental data mining, we identified both known and novel possible host-virus pairs. In particular, the growth of prokaryotes associating with phytoplanktonic organic matter, such as Bacteroidetes (*Polaribacter* and NS9 marine group), *Vibrio* spp., and Rhodobacteriales (*Nereida* and *Planktomarina*), was accompanied by an increase in viruses predicted to infect Bacteroidetes, *Vibrio*, and Rhodobacteriales, respectively. Collectively, our findings suggest that changes in bloom-forming species can be followed by an increase in a specific group of prokaryotes and their viruses and that elucidating these tripartite relationships among specific phytoplankton, prokaryotes, and prokaryotic viruses improves our understanding of coastal biogeochemical cycling in blooms.

**IMPORTANCE:**

The primary production during marine phytoplankton bloom and the consumption of the produced organic matter by heterotrophic prokaryotes significantly contribute to coastal biogeochemical cycles. While the activities of those heterotrophic prokaryotes are presumably affected by viral infection, the dynamics of their viruses during blooms are not fully understood. In this study, we experimentally demonstrated that intracellular fractions of taxonomically distinct bloom-forming phytoplankton species, the diatom *Chaetoceros* sp. and the raphidophycean alga *Heterosigma akashiwo,* promoted the growth of taxonomically different prokaryotes and prokaryotic viruses. Based on their dynamics and predicted hosts of those viruses, we succeeded in detecting already-known and novel possible host-virus pairs associating with either phytoplankton species. Altogether, we propose that the succession of bloom-forming phytoplankton would change the composition of the abundant prokaryotes, resulting in an increase in their viruses. These changes in viral composition, depending on bloom-forming species, would alter the dynamics and metabolism of prokaryotes, affecting biogeochemical cycling in blooms.

## INTRODUCTION

Marine dissolved organic matter (DOM) is one of the major carbon pools on Earth (1, 2). Phytoplankton are mainly responsible for marine primary production, which is known to be comparable to terrestrial levels, and are the major source of marine DOM (3). Some species like *Heterosigma akashiwo* (Raphidophyceae) and *Chaetoceros* spp. (Diatomea) eventually form blooms in coastal areas through local and seasonal massive growth (4–7). Phytoplankton blooms are often constituted by multiple species (4, 8), and the organic matter produced by each species varies (9–12). Thus, DOM released during blooms comprises complex mixtures of a vast range of different organic molecules (4).

A large portion (10%–50%) of the DOM pools produced and released by phytoplankton is consumed and converted into smaller molecules by heterotrophic prokaryotes (13). Recent metagenomic and metaproteomic studies in natural blooms have revealed that the relative abundance of specific prokaryotic taxa, such as Flavobacteriales (Bacteroidia), Rhodobacteriales (Alphaproteobacteria), Alteromonadales (Gammaproteobacteria), and Oceanospirales (Gammaproteobacteria), which are capable of utilizing DOM released from phytoplankton, increased drastically within a week (4, 5, 14, 15). Based on microcosm experiments, patterns of shifts in taxonomic composition and diversity of prokaryotic communities were suggested to depend on bloom-forming phytoplankton species (11, 12, 16). This is explained by the fact that DOM production varies among phytoplankton species and utilizable and preferred organic molecules vary among prokaryotes (10, 17). One of those studies further reported that shifts in the prokaryotic community led to changes in amino acid composition (12), indicating that the DOM is actually consumed and converted by heterotrophic prokaryotes. Accordingly, the phytoplanktonic composition in blooms affects the heterotrophic prokaryote community structure and the resultant biogeochemical cycles (4).

In addition to their relationships with phytoplankton, the ecology of these heterotrophic prokaryotes and the resultant biogeochemical cycles are affected by other biotic interactions in the natural environment (18–20). In particular, viruses are the most abundant biological entities, and many infect marine prokaryotes (21–23). A theoretical study estimated that viral infection of prokaryotes contributes to 10%–20% of prokaryotic cell mortality per day (24), and a most recent study analyzing prokaryotic and viral dynamics in a coastal seawater reported that most abundant prokaryotic taxa are frequently infected in the natural environment (25). Those viral infections enable the release of their cellular compounds and metabolites into DOM pools, which can be readily taken up by other microorganisms (21–23). Some prokaryotic viruses also modulate host metabolism for efficient infection through genes called viral auxiliary metabolic genes (AMGs), which are evolutionarily derived from their host genomes (26, 27). Therefore, to understand microbial ecology involving biogeochemical cycles in natural blooms formed by multiple phytoplankton species, their effect on the dynamics of prokaryotic viruses also needs to be elucidated. A previous study conducting microscopic observations of viral particles suggested that viral production is active during natural phytoplankton blooms (28). Furthermore, a recent study combining isolation techniques and metagenomic analysis found that taxonomically diverse Flavobacteriales viruses emerged during a natural bloom of mostly diatoms (29). However, the ecological and taxonomic links between bloom-forming phytoplankton, marine heterotrophic prokaryotes, and prokaryotic viruses remain unclear.

In the present study, we hypothesized that shifts in prokaryotic community corresponding to bloom-forming species facilitate infection and increase of distinct prokaryotic viruses. Thus, we observed the effects of DOM released from phytoplankton on prokaryotic community shifts and dynamics of prokaryotic viruses. We performed a microcosm experiment in which the dynamics of coastal prokaryotic and viral communities were analyzed by 16S rRNA gene amplicon and viral metagenomic analyses, respectively, during incubation under laboratory conditions with intracellular fractions obtained from cultured strains of bloom-forming species *H. akashiwo* and *Chaetoceros* sp. for following reasons: (i) these are both globally distributed species and thus biogeochemically important (5–7, 30–33). (ii) These often occur in the same localities, such as Osaka Bay, Japan, and the San Pedro Channel, USA (5, 6, 34). (iii) These are suitable to clarify the differences in the impact on the prokaryotic viral community. Previous studies revealed distinct prokaryotic responses to each species: organic matters from *H. akashiwo* stimulate the increase of Alteromonadales and Vibrionales (35), while those from diatoms promote that of Flavobacteriales (14, 15, 36). These differences would facilitate testing our hypothesis. Furthermore, we analyzed the environmental metagenomic data obtained from Osaka Bay, Japan (25), to confirm whether the prokaryotic and viral dynamics observed in our experiment occurred in the natural environment. We propose that different phytoplankton blooms lead to distinct dynamics of prokaryotic viruses and can differentially affect biogeochemical cycling.

## RESULTS

### Abundance shifts of prokaryotic cells and viral particles in microcosms

**Fig 1:**
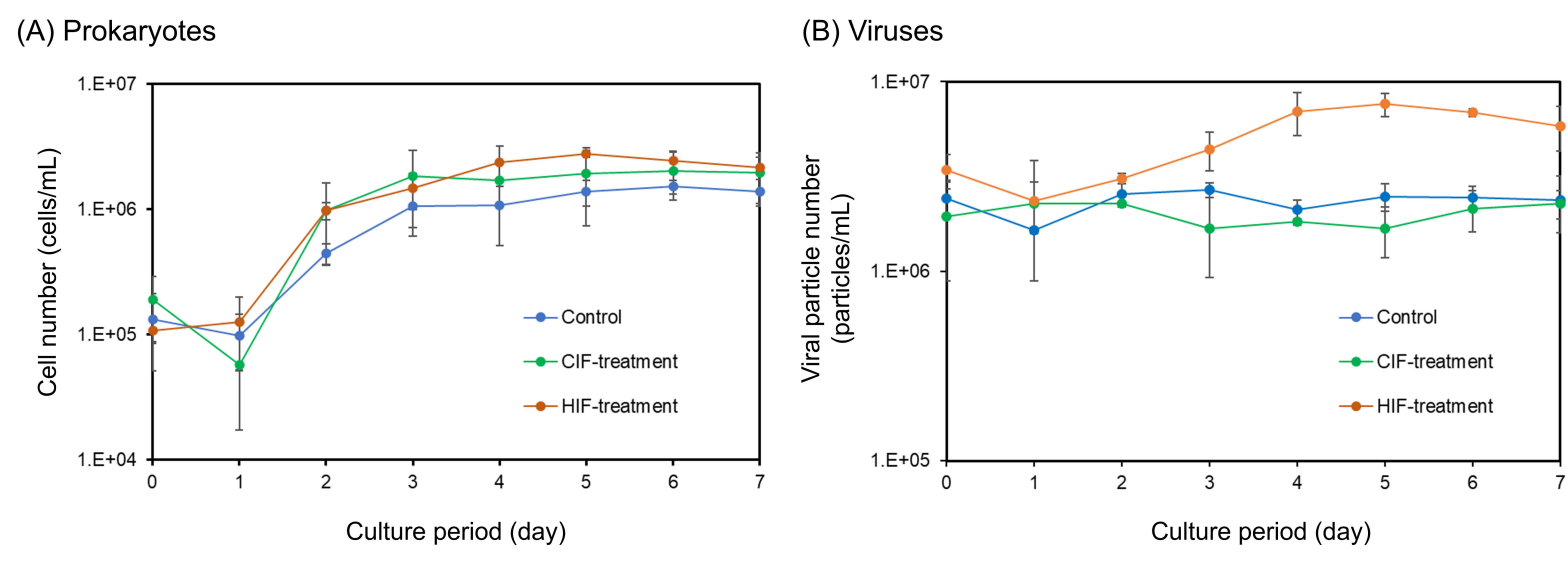
Shifts in the abundance of (**A**) prokaryotic cells and (**B**) viral particles during the microcosm experiment. Cell and viral counts were obtained using flow cytometry. The average cell and viral numbers in the triplicate flasks are shown. Error bars indicate standard deviation. Control: samples cultured without any additional organic matter. CIF treatment: samples cultured with CIF. HIF treatment: samples cultured with HIF.

First, we investigated the effects of dissolved intracellular fractions of *Chaetoceros* sp. and *H. akashiwo* (CIF and HIF, respectively) on the growth of prokaryotes in the natural seawater of Osaka Bay. Cell density in all treatments showed similar dynamics and abundance (Fig. 1A). While the cell densities decreased (the control and CIF treatment) or slightly increased (HIF treatment) from day 0 to day 1, all the treatments reached their maximum (1.52 ± 0.18 × 10^6^–2.78 ± 0.19 × 10^6^ cells/mL) by day 6 followed by stagnation. Based on cell density dynamics, we defined days 0–1 as the “early phase” when cells were decreasing or slightly increasing, days 2–4 as the “middle phase” when cells were increasing, and days 5–7 as the “late phase” when cell densities were almost saturated. While the cell densities of both the CIF and HIF treatments were higher than those of the control in the middle phase, only the difference in cell densities between the HIF treatments and the control in the late phase became statistically significant (Mann–Whitney *U* test: *P* < 0.01). In both CIF and HIF treatments, the cell densities were higher in the control, being consistent with a previous study indicating an increase in prokaryotes during bloom periods (28). However, a significant increase is observed only in the HIF treatments, suggesting that the extent of prokaryotic growth differs depending on bloom-forming species. This corresponds with the previous observation mentioning that the degree of prokaryotic production varies among phytoplanktonic species (37).

While the viral abundance of control and CIF treatments was stable during the whole culture period (Fig. 1B; 2.34 ± 0.38 × 10^6^ particles/mL in control and 2.01 ± 0.50 × 10^6^ particles/mL in CIF treatment, on average), that of HIF-treatment samples increased from day 1 to day 5 (2.36 ± 1.47 × 10^6^–7.63 ± 1.06 × 10^6^ particles/mL). The viral particle abundance in HIF treatments was significantly greater than that in the control and CIF treatment during the middle and late phases (Mann–Whitney *U* test: *P* < 0.01). The abundance of these prokaryotic cells and viral particles was used to estimate the absolute dynamics of particular taxa in subsequent analyses.

Additionally, when we compared the abundance of prokaryotes and viruses in each treatment, both CIF and HIF treatments showed a similar trend wherein an increase in prokaryotic abundance is followed by an increase in viral abundance except for the early phase in CIF treatment (Fig. S1). These results agree well with a previous report that production levels of both prokaryotes and viruses concurrently increase in natural bloom (28).

### Shifts in prokaryotic community structure in response to organic matter

**Fig 2:**
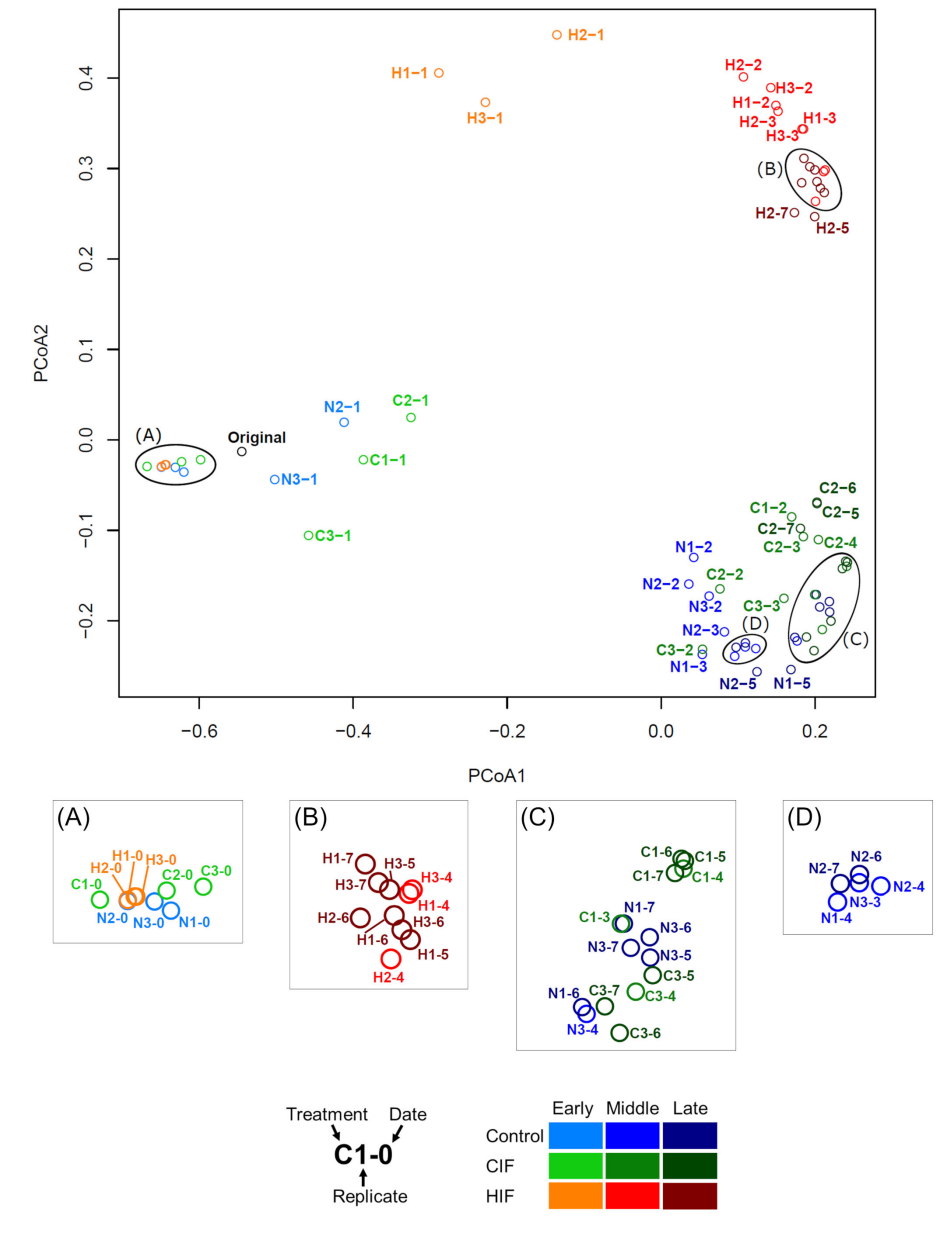
Comparison of ASV compositions among control, CIF, and HIF treatments. The number of sequences of each sample was rarified into 9,450 reads prior to the analyses. Bray–Curtis dissimilarity among all samples was illustrated by PCoA. Samples are distinguished by colors based on the treatments and culture periods. Enlarged figures of areas (**A**)–(**D**) are shown separately.

Next, we performed 16S rRNA gene amplicon sequencing analysis to confirm the taxonomic difference of the marine prokaryotic community triggered by the intracellular fractions obtained from different phytoplankton species. A total of 415 amplicon sequence variants (ASVs) were obtained from the original seawater sample. During the microcosm experiments, 1,017 ASVs were obtained in the control and 1,049 and 743 ASVs were obtained on average per flask in CIF and HIF treatments, respectively (Table S1). The difference in ASV composition during cultivation was visualized using principal coordinate analysis (PCoA) based on Bray–Curtis dissimilarity (Fig. 2). While all the samples on day 0 were closely plotted with the original seawater sample, the samples in the middle and late phases were clustered by treatment, separately from their early phases [analysis of similarity (ANOSIM), *P* < 0.01]. The PCoA indicated that prokaryotic community structures explicitly shifted prior to the middle phase, depending on (i) the presence or absence of the phytoplanktonic dissolved intracellular fraction and (ii) the taxonomic difference in phytoplanktonic as the source of the fraction.

Thus, we analyzed the dynamics of dominant phyla (class level for Proteobacteria) in each treatment (Fig. S3). All the samples on day 0 were dominated by Alphaproteobacteria and Gammaproteobacteria (27.1%–28.8% and 28.8%–34.1%, respectively), followed by Bacteroidetes (18.6%–23.0%). After day 0, these three bacterial groups were still dominant, but their proportions differed among the treatments, supporting the PCoA plotting patterns (Fig. 2).

### Dynamics of abundant ASVs in response to organic matter

We focused on the growth of prokaryotes at the ASV level for each treatment. Since increases and decreases in relative abundance during cultivation do not necessarily indicate cell growth and death, we analyzed the dynamics of abundant ASVs based on the approximate cell number, which was calculated with the total cell number and relative abundance, as described in the Materials and Methods section.

**Fig 3:**
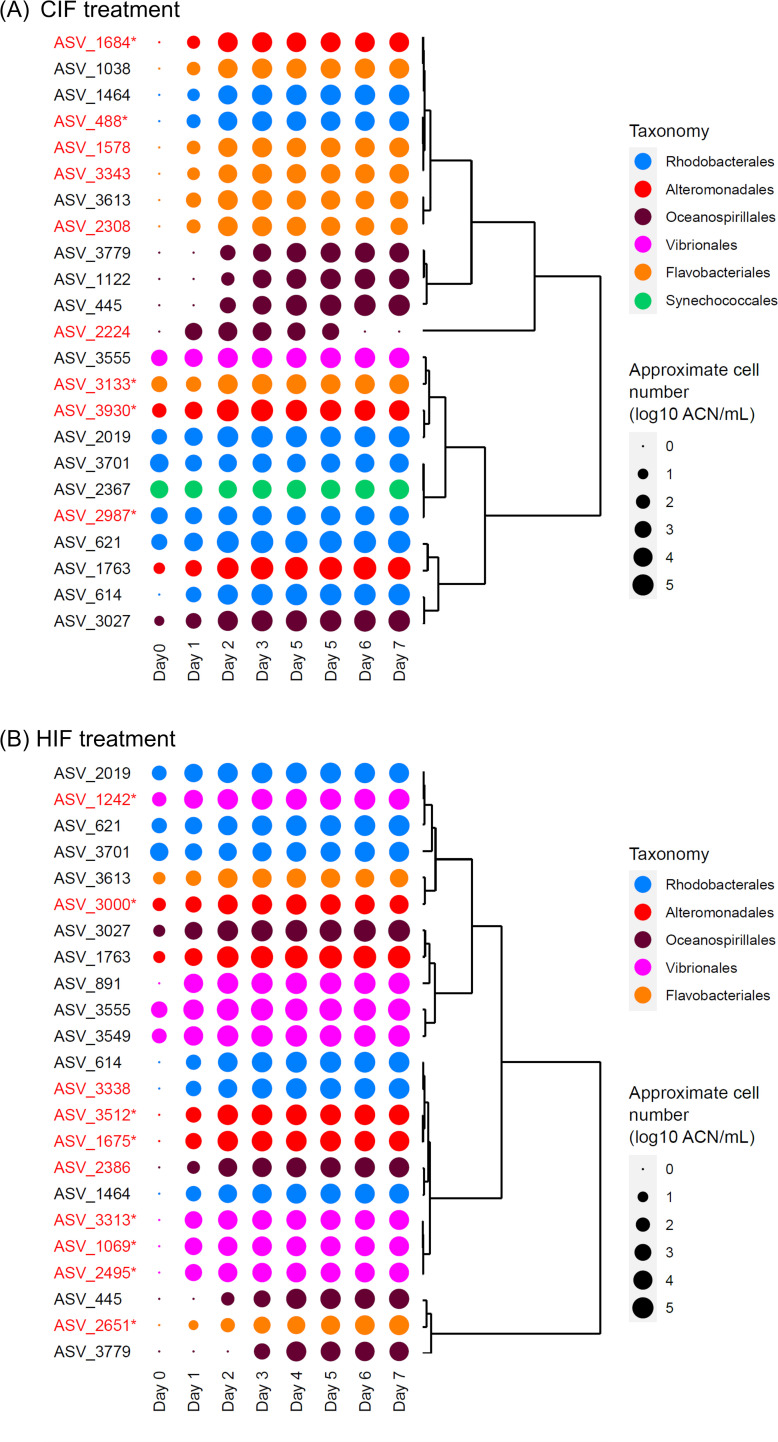
Dynamics of ASVs which were found to be abundant in (**A**) CIF and (**B**) HIF treatments. Averages of the approximate cell number in the triplicate flasks are shown as plots in the log scale. The plot colors show the order-level taxonomy of each ASV. Dendrograms represent the similarity of dynamics among ASVs. ASVs abundant only in the CIF or HIF treatments are highlighted in red, and if they are treatment-specific ASVs, they are indicated by asterisks.

We obtained 18, 23, and 23 abundant ASVs in the control, CIF, and HIF treatments, respectively. Sixteen of these ASVs were shared between two or three treatments (Fig. S4; Table S5). Among these, eight ASVs that became abundant in all treatments were capable of growing independently from phytoplanktonic intracellular fractions (Fig. 3; Fig. S5). Three ASVs that were found to be abundant in both the CIF and HIF treatments were regarded as those that were capable of thriving by using either intracellular fraction.

A total of 9 and 10 ASVs passed the criterion of abundant ASVs in either CIF or HIF treatments, respectively (Fig. S4; Table S5). To confirm whether these ASVs were specifically abundant in the CIF or HIF treatments, we performed a differential abundance test using LEfSe. As a result, five and eight ASVs were significantly more abundant in either CIF or HIF treatments than in the other treatments, respectively. As these were hypothesized to respond to the CIF or HIF, we referred to them as CIF-specific and HIF-specific ASVs, respectively (Table 1). According to the approximate cell number, the CIF- and HIF-specific ASVs detected above were suggested to start growing within 1 day from day 0 (Fig. 3), followed by saturation by the end of the middle phase.

**TABLE 1:** Taxonomic assignment of CIF- and HIF-specific ASVs^*a*^

ASV ID		Taxonomy			Confidence	Top hit organism (accession number)
		Order	Family	Genus		
CIF specific	ASV_1684	Alteromonadales	Alteromonadaceae		1.000	
	ASV_2987	Rhodobacterales	Rhodobacteraceae	*Planktomarina*	0.998	*Planktomarina temperata* RCA23 (NR_117309, NR_125550)
	ASV_3133	Flavobacteriales	Flavobacteriaceae	*Polaribacter*	0.987	
	ASV_3930	Alteromonadales	Flavobacteriaceae	*Glaciecola*	0.858	*Glaciecola amylolytica* strain THG-3.7 (NR_171501)
	ASV_488	Rhodobacterales	Rhodobacteraceae	*Nereida*	0.981	*Nereida ignava* strain CECT5292 (NR_115897)
						*Nereida ignava* strain 2SM4 (NR_042283)
HIF specific	ASV_1069	Vibrionales	Vibrionaceae	*Vibrio*	0.837	*Vibrio splendidus* strain LMG 4042 (NR_114888)
	ASV_1242	Vibrionales	Vibrionaceae	*Vibrio*	0.763	
	ASV_1675	Alteromonadales	Pseudoalteromonadaceae	*Pseudoalteromonas*	1.000	
	ASV_2495	Vibrionales	Vibrionaceae	*Vibrio*	0.982	
	ASV_2651	Flavobacteriales	NS9 marine group	NS9 marine group	0.920	
	ASV_3000	Alteromonadales	Shewanellaceae	*Psychrobium*	0.939	
	ASV_3313	Vibrionales	Vibrionaceae	*Vibrio*	0.978	*Vibrio gigantis* strain LGP 13 (NR_114910)
	ASV_3512	Alteromonadales	Pseudoalteromonadaceae	*Pseudoalteromonas*	0.704	*Pseudoalteromonas marina* strain mano4 (NR_042981)

^*a*^ Regarding ASVs whose sequence showed 100% identity with cultured bacteria deposited in the RefSeq NR database, the hit bacterium is indicated with the corresponding accession number.

### Taxonomic characteristics of CIF- and HIF-specific ASVs

The taxonomic compositions of CIF- and HIF-specific ASVs were different at the genus level. The CIF-specific ASVs (Table 1) included *Nereida* (ASV_488, Rhodobacteriales), *Planktomarina* (ASV_3930, Rhodobacteriales), Alteromonadaceae (ASV_1684, genus unassigned), *Glaciecola* (ASV_3930, Alteromonadales), and *Polaribacter* (ASV_3133, Flavobacteriales). ASV_488, ASV_2987, and ASV_3930 showed 100% identity of nucleotide sequences with marine bacteria *Nereida ignava* (NR_115897 and NR_042283), *Planktomarina temperata* (NR_117309 and NR_125550), and *Glaciecola amylolytica* (NR_171501), respectively (Table 1). These species or families are known to be associated with diatom blooms or respond to the organic matter of diatoms (4, 10, 14, 15, 38–40).

In contrast, the HIF-specific ASVs were classified into the genera *Psychrobium* (ASV_3000, Alteromonadales), *Pseudoalteromonas* (ASV_1675 and ASV_3512, Alteromonadales), *Vibrio* (ASV_1069, ASV_1242, ASV_2495, and ASV_3313, Vibrionales), and NS9 marine group (ASV_2651, Flavobacteriales) (Table 1). Of these, ASV_3512, ASV_1069, and ASV_3313 had 100% nucleotide sequence identity with *Pseudoalteromonas marina* (NR_042981), *Vibrio splendidus* (NR_114888), and *Vibrio gigantis* (NR_114910), respectively (Table 1); *V. splendidus* causes mortality in mussels and oysters (41, 42). *P. marina* and the genus *Vibrio* appeared to respond to the organic matter of *H. akashiwo* in our previous microcosm experiments (35). Although not specific to *H. akashiwo*, the abundance of *Psychrobium* and NS9 marine groups of uncultured Flavobacteriales members is also known to be associated with the dynamics of marine phytoplankton in natural environments (43, 44).

The taxonomic composition of the CIF- and HIF-specific ASVs suggests that the dissolved intracellular fractions of different phytoplanktonic species promote bloom-responding bacteria belonging to distinct genera.

### Dynamics of prokaryotic viruses following shifts in prokaryotic communities

**Fig 4:**
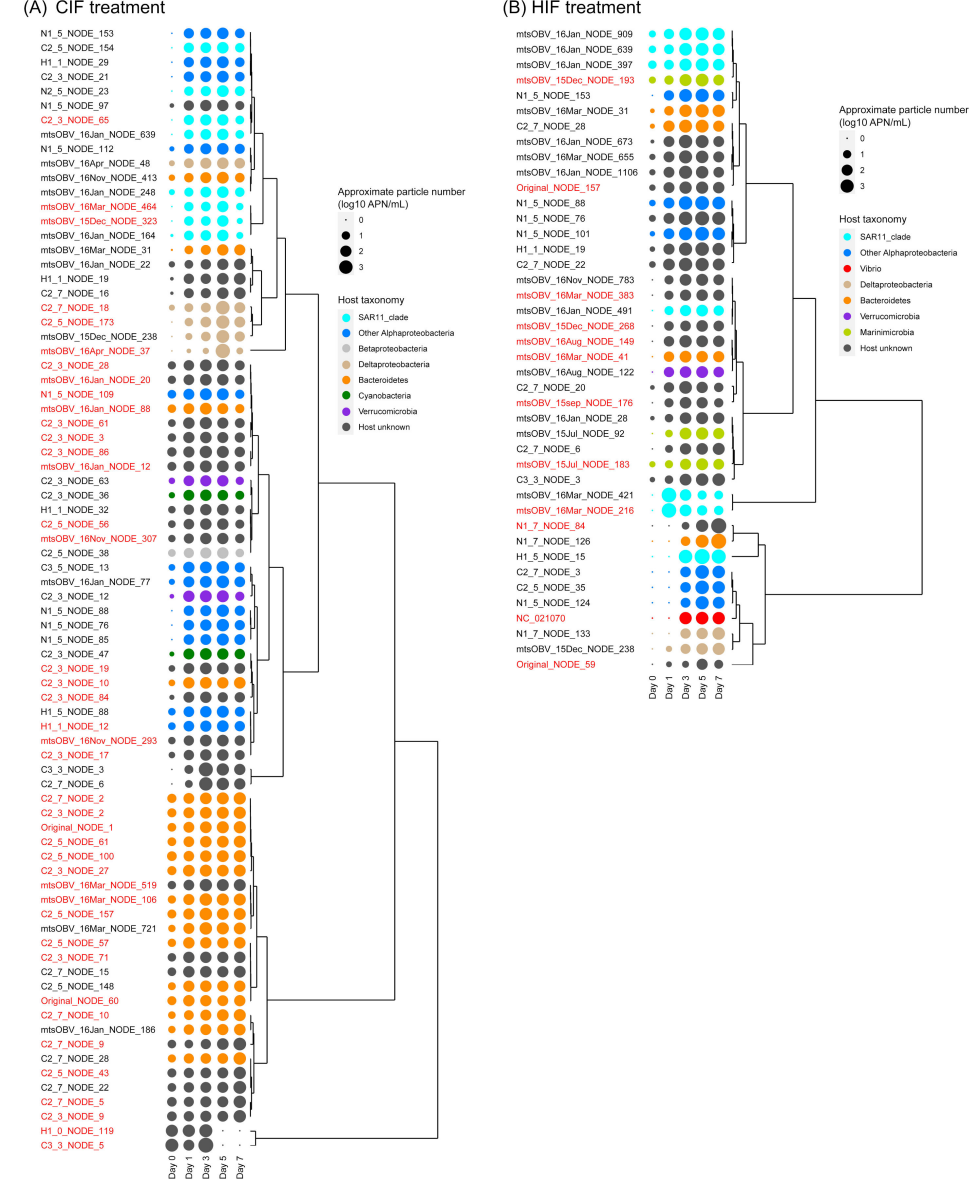
Dynamics of vOTUs that significantly increased in (**A**) CIF and (**B**) HIF treatments. Averages of approximate particle numbers in the triplicate flasks are shown by plots in the log scale. The plot colors show the taxonomy of the putative host. Dendrograms represent the similarity of dynamics among vOTUs. Treatment-specific vOTUs are highlighted in red.

Given our finding that CIF and HIF have different effects on the prokaryotic community structures and growth of particular prokaryotic taxa, we investigated the viruses present in the microcosm cultures. To detect viruses that explicitly increased during the experiment in each treatment, we first prepared a virome data set consisting of 9,313 non-redundant viral operational taxonomic units (vOTUs) (>10 kb), of which 465 were newly assembled in this study (Tables S3 and S4), while 8,848 were collected from the previous studies (25, 45). We calculated the approximate particle numbers of these viruses in our microcosm samples. Since the majority of the abundant prokaryotic ASVs increased from day 1 (see above, Fig. 3), we regarded vOTUs, which significantly increased after day 1 compared to day 0 in the approximate particle number as the increased vOTUs associating with the abundant ASVs. As a result, 99, 78, and 42 vOTUs passed this criterion in the control, CIF, and HIF treatments, respectively (Fig. S6; Table S6). Fifty-five vOTUs were significantly increased in at least two treatments. Six vOTUs that increased in all treatments were considered to be increasing, not relating to the phytoplanktonic intracellular fractions (Fig. 4; Fig. S7). Five vOTUs that increased in both CIF and HIF treatments were regarded as vOTUs associated with both phytoplanktonic intracellular fractions. In contrast, 41 and 13 vOTUs significantly increased only in either CIF or HIF treatments, respectively (Fig. S6; Table S6). We named these vOTUs CIF- and HIF-specific vOTUs, respectively. Note that the definition of the “specific vOTUs” is different from that of the “specific ASVs”; while specific ASVs showed significant specificity to CIF or HIF in its abundance, specific vOTUs showed significant increase from day 0 in response to CIF or HIF. Given these specific vOTUs, different prokaryotic viruses increased when different phytoplankton-derived intracellular fractions were added.

Of the 41 CIF-specific vOTUs, we succeeded in predicting the prokaryotic hosts for 21 (Tables S3 and S6). Most of them were predicted to be Bacteroidetes viruses (13 vOTUs). The rest belonged to SAR11 clade viruses (three vOTUs), Deltaproteobacteria viruses (three vOTUs), and non-SAR11 clade Alphaproteobacteria viruses (two vOTUs; Rhodobacteriales virus and Rhodospirillales virus) (Tables S4 and S6). For taxonomic classification of the vOTUs, we assigned them to genomic operational taxonomic units (gOTUs), which are genus-level classification of viral genomes. In summary, these vOTUs were classified into 11 gOTUs (Table S6). Notably, three host unknown vOTUs were labeled as lysogenic viruses (Table S6).

The 13 Bacteroidetes vOTUs belonged to g405, g481, g506, g509, or g515 at the gOTU level, which constitutes one of the most abundant viral groups in the ocean, group 2 Bacteroidetes viruses (46). As described above, *Polaribacter* was the only Bacteroidetes (order Flavobacteriales) bacterium that appeared to grow specifically in CIF treatment (Table 1). Notably, the 13 Bacteroidetes viruses did not show sufficiently high similarity in amino acid sequences (Sg < 0.15) with any cultured *Polaribacter* viruses (five strains: *Polaribacter* phage P12002S, P12002L, Danklef_1, Freya_1, and Leef_1; NC_028763, NC_028924, NC_062745, NC_062746, and NC_062750, respectively). In addition, 3 of the 13 viruses encoded glycoside hydrolase GH16, a CAZyme that mainly hydrolyzes β-1,3-glucosyl linkages of β-glucan, such as laminarinase (47) (Table S7). This AMG is not present in any genomes of the isolated *Polaribacter* viruses (29, 48) and previously published group 2 Bacteroidetes viruses (25, 45).

Additionally, the CIF-specific ASVs included Rhodobacteriales bacteria, *Nereida,* and *Planktomarina* (Fig. 3; Table 1), consistent with the existence of CIF-specific Rhodobacteriales vOTU (H1_1_NODE_12). Although Alteromonadales (Gammaproteobacteria) ASVs were detected as CIF specific (see above), no CIF-specific vOTU was assigned to an Alteromonadales or Gammaproteobacteria virus (Fig. 4A).

We also succeeded in identifying the hosts for 6 of the 13 HIF-specific vOTUs (Tables S3 and S6), including two SAR11 clade viruses, two Marinimicrobia viruses, one Gammaproteobacteria virus, and one Bacteroidetes virus. The Gammaproteobacteria virus was previously isolated as a virus that infects *Vibrio splendidus* (Vibrio phage martha 12B12; NC_021070). These vOTUs were assigned for six gOTUs (Table S6). Contrary to the CIF-specific vOTUs, no lytic viruses were identified among the HIF-specific vOTUs. As the transition to the lytic cycle of lysogenic viruses is triggered by environmental changes to the host, the addition of CIF is likely to exert drastic effects on certain prokaryotes (23).

*Vibrio* ASVs were detected as HIF specific, including *V. splendidus* (Fig. 3; Table 1). In addition, the HIF-specific ASVs included the NS9 marine group (Bacteroidetes), consistent with the host prediction of the HIF-specific Bacteroidetes vOTU. It is noteworthy that unlike some CIF-specific Bacteroidetes vOTUs, we could not find any AMG in the genomes of the HIF-specific vOTUs. Similar to CIF treatment, HIF-specific vOTUs assigned to Alteromonadales viruses were not detected, although the HIF-specific ASVs included Alteromonadales ASVs (Fig. 4B; Table S6).

Although taxonomically different SAR11 vOTUs were detected as CIF- and HIF-specific vOTUs (Fig. 4; Table S6), no SAR11 ASV was identified as an abundant ASV. Since four SAR11 ASVs belonging to different clades (clades Ia: three ASVs and clade II: one ASV) were abundant in the original seawater sample (Table S8), the detected SAR11 vOTUs might be derived from an association with any of the SAR11 ASVs. Additionally, although Rhodospirillales vOTU and Deltaproteobacteria vOTUs were identified as CIF-specific and Marinimicrobia vOTUs were detected as HIF specific (Fig. 4; Table S6), any abundant ASVs both in the original seawater and the microcosm samples were not annotated as the three taxonomic groups of Rhodospirillales, Deltaproteobacteria, and Marinimicrobia (Tables S5 and S8), suggesting that the viruses might also infect prokaryotes of another lineage.

### Co-occurrence of treatment-specific ASVs and vOTUs

Here, we focused on 16 of the treatment-specific vOTUs predicted to infect Rhodobacteriales, *Vibrio*, and Bacteroidetes, as the host prokaryotic groups of those vOTUs were detected as specific ASVs in the same treatments. (Table 2). We examined the consistency between the dynamics of the vOTUs and those of the ASVs in our microcosm samples. In the CIF treatment, the Bacteroidetes *Polaribacter* ASV and 13 group 2 Bacteroidetes vOTUs grew until day 3 (Fig. 3A and 4A). Rhodobacteriales *Nereida* and *Planktomarina* ASVs grew until day 2, and the Rhodobacteriales vOTU increased from day 0 to day 1. The abundance of the ASVs and the vOTU saturated by the end of the middle phase (Fig. 3A and 4A). In the HIF treatment, the four *Vibrio* ASVs grew until day 3, followed by saturation. Similarly, the *Vibrio* vOTU increased until day 3 (Fig. 3B and 4B). The Bacteroidetes NS9 ASV, gradually grew from day 0 to day 7, while the Bacteroidetes vOTU increased until day 3, followed by saturation (Fig. 3B and 4B). Collectively, 16 Rhodobacteriales, *Vibrio*, and Bacteroidetes vOTUs showed co-occurrence dynamics with prokaryotic ASVs with the corresponding taxonomy; the vOTUs increased at the same time as or soon after the growth of the prokaryotic ASVs.

**TABLE 2:** Co-occurrence pairs between CIF-specific ASVs-vOTUs and HIF-specific ASVs-vOTUs

ASV and vOTU type and predicted pairs	ASV ID	Taxonomy	vOTUs	gOTU
CIF specific				
Bacteroidetes host-virus pairs	ASV_3133	*Polaribacter*	C2_3_NODE_10	509
			C2_3_NODE_2	515
			C2_3_NODE_27	509
			C2_5_NODE_100	515
			C2_5_NODE_157	506
			C2_5_NODE_57	509
			C2_5_NODE_61	515
			C2_7_NODE_10	509
			C2_7_NODE_2	515
			Original_NODE_1	515
			Original_NODE_60	481
			mtsOBV_16Jan_NODE_88	405
			mtsOBV_16Mar_NODE_106	506
Alphaproteobacteria host-virus pairs	ASV_2987	*Planktomarina*	H1_1_NODE_12	389
	ASV_488	*Nereida*		
HIF specific				
Bacteroidetes host-virus pairs	ASV_2651	NS9 marine group	mtsOBV_16Mar_NODE_41	504
*Vibrio* host-virus pairs	ASV_1069	*Vibrio*	NC_021070	1,034
	ASV_1242	*Vibrio*		
	ASV_2495	*Vibrio*		
	ASV_3313	*Vibrio*		

**Fig 5:**
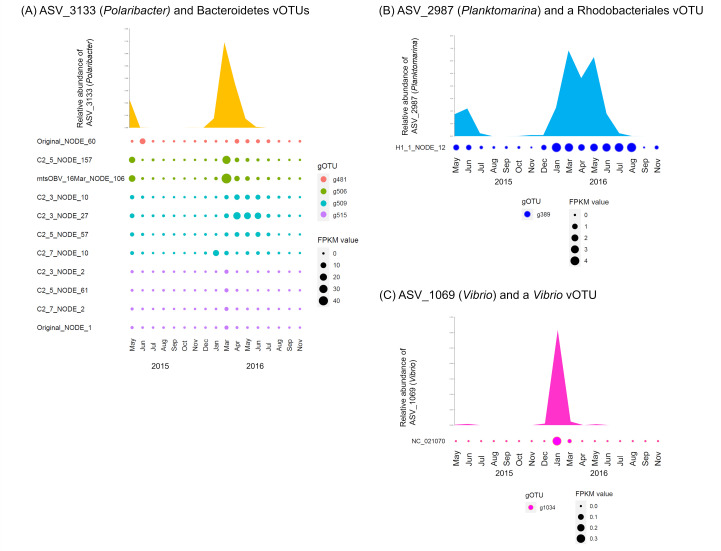
Co-occurrence dynamics of the marine prokaryote viruses and prokaryotes in the Osaka Bay natural seawater samples. (**A**) ASV_3133 (*Polaribacter*) and Bacteroidetes vOTUs. (**B**) ASV_2987 (*Planktomarina*) and a Rhodobacteriales vOTU. (**C**) ASV_1069 (*Vibrio*) and a *Vibrio* vOTU. The environmental data sets collected monthly between May 2015 and November 2016 (Tominaga et al*.*, in press) (25) were used. Relative abundance of each ASV was calculated by mapping quality-controlled reads of 16S rRNA genes to the ASV sequence with 100% identity using VSEARCH. Fragments per kilobase per mapped million reads (FPKM) value in each vOTU was calculated by mapping quality-controlled viral metagenomic reads to the sequence of the vOTU with 95% identity using Bowtie2. Co-occurrence dynamics are shown only if a significantly positive correlation is detected (Spearman correlation; *r* > 0.6, *P* < 0.01, and *q* < 0.05).

To determine whether the above co-occurrence dynamics of these vOTUs and prokaryotic ASVs could be observed in the environment, we performed a correlation network analysis between them using the 17 data sets from Osaka Bay, collected monthly between 2015 and 2016 (25) (Table S9). We found a significant positive correlation in 13 cases (*r* > 0.6, *P* < 0.01, and *q* < 0.05) (Table S10). The *Polaribacter* ASV was detected from May to June 2015, increased from January to March 2016, and decreased again by June. The 11 Bacteroidetes vOTUs peaked in May–June 2015 or January–June 2016, though dynamic patterns were diverse among the vOTUs (Fig. 5A). The *Planktomarina* ASV showed similar dynamics to the Rhodobacteriales vOTU, exhibiting an increase in May–June 2015 and December 2015–August 2016 (Fig. 5B). Similarly, the *V. splendidus* and the *Vibrio* vOTU increased in January 2016 (Fig. 5C).

In addition, as we did not detect Alteromonadales viruses as treatment-specific vOTUs in our microcosm experiments, we subjected the host-unknown, CIF-, and HIF-specific vOTUs (Table S6), and treatment-specific Alteromonadales ASVs to the same analyses. In the CIF treatment, 14 host-unknown vOTUs started to increase simultaneously with the CIF-specific Alteromonadales ASVs (Table S11; Fig. 3A and 4A). Similarly, host-unknown HIF-specific vOTUs increased during the growth of HIF-specific Alteromonadales ASVs (Table S11; Fig. 3B and 4B). Of these, three of the co-occurrence pairs found in the HIF treatment showed significant correlative dynamics (*r* > 0.6, *P* < 0.01, and *q* < 0.05) in the natural seawater samples (Table S10): ASV_1675, ASV_3512 (*Pseudoalteromonas*)-Original_NODE_59, and ASV_3000 (*Psychrobium*)- mtsOBV_16Mar_NODE_383 (Fig. S8). The eight genes in the Original_NODE_59 genome, which have functionally annotated homologs in cellular organisms or viruses, were all similar to the genes present in Alphaproteobacteria virus genomes (GHOSTX; *e*-value < 1E-8) (Table S12). For instance, the amino acid sequences of proteins encoded in gene1 and gene10 of this vOTU were highly similar to the putative tail fiber protein of *Pelagibacter* phage HTVC033P (MZ892993_66; *e*-value = 1.4E-44) and the putative terminase small subunit of *Roseobacter* phage CRP-212 (MZ892988_42; *e*-value = 3.9E-27). In contrast, genes of mtsOBV_16Mar_NODE_383 had homologs in taxonomically diverse bacteria and viruses (GHOSTX; *e*-value < 1E-8) (Table S12), and the amino acid sequences of proteins encoded in gene5 and gene10 were similar to those of PD-(D/E)XK nuclease-like domain-containing protein of *Mycobacterium heckeshornense* (WP_071700202, *e*-value = 6.90E-11) (Actinobacteria) and DNA cytosine methyltransferase of *Margalitia camelliae* (WP_101355680, *e*-value = 3.8E-51) (Bacillota), respectively. Amino acid sequences of proteins encoded by gene21 and gene25 of mtsOBV_16Mar_NODE_383 were similar to those of a phage terminase large subunit of *Zunongwangia* phage (WP_084841783, *e*-value = 1.5E-44) and a phage tail tape measure protein of *Lutibacter* phage (WP_144895146, *e*-value = 3.6E-27), both of which are viruses infecting the class Bacteroidia (phylum Bacteroidetes).

## DISCUSSION

In the present study, combining 16S rRNA gene amplicon and viral metagenomic analyses in a microcosm experiment, we demonstrated two important aspects of microbial ecosystems in marine environments: (i) specific prokaryotic viruses can increase following changes in the prokaryotic community within a few days and (ii) their composition was affected by the bloom-forming phytoplanktonic species. So far, those relational dynamics among certain phytoplanktonic species, prokaryotes, and prokaryotic viruses in natural bloom were proposed by dynamics-based network analyses (49). However, the previous study focused on only a specific viral lineage (i.e., T4-like-myoviruses), and the host information of those viruses was not revealed (49). Therefore, the dynamics of the prokaryotic virus community during different blooms remain incompletely understood, and how the dynamics of these viruses associate with those of their hosts is still to be revealed. Another recent study found a tight link between the increase of Flavobacteriales viruses and natural diatom bloom by isolation techniques and metagenomic analysis (29), leaving a concern whether the increase of Flavobacteriales viruses is specific to the diatom blooms. In this study, we revealed the effects of intracellular fractions derived from different phytoplanktonic species on prokaryotes and prokaryotic viruses by community-wide analysis with the host information of those viruses. Thus, we could elucidate the specific relationships among particular phytoplankton, prokaryotes, and their infecting prokaryotic viruses. Although our study still has not been able to validate the entire changes in prokaryotic viral dynamics corresponding to those of different phytoplankton species in natural blooms, we confirmed the particular host-virus pairs predicted in our experiment co-occurred in the natural environment as well. Furthermore, functional annotation analyses allowed us to speculate the effects of different viral dynamics corresponding to bloom-forming species on biogeochemical cycling.

We first confirmed that the addition of dissolved intracellular fractions derived from different phytoplanktonic species promotes the growth of taxonomically distinct prokaryotes that are known to react to phytoplanktonic organic matter or are associated with blooms. The differentially grown prokaryotes in different phytoplankton-derived intracellular fractions (Fig. 3; Table 1) had an associated increase in distinct prokaryotic viruses (Fig. 4). As the treatment-specific viruses were rarely detected on day 0 in this study (Fig. 4), these viruses likely increased in abundance by infecting dominant prokaryotes; if the kill-the-winner hypothesis stands, prokaryotic taxa with a faster growth rate or higher abundance are more susceptible to viral infection (50). Thus, we succeeded in identifying both known and new possible host-virus pairs in our microcosm experiment (Table 2).

The host-virus pair identified with most certainty was *V. splendidus* ASV_1069 and its virus Vibrio phage martha 12B12 (NC_021070), which appeared in the HIF treatment (Fig. 3 and 4; Table 2). The co-occurrence between them in the Osaka Bay samples further supports the *V. splendidus*-Vibrio phage martha 12B12 relationship (Fig. 5C; Table S10). The preference of *Vibrio* spp. for the intracellular component of *H. akashiwo* was in good agreement with our previous study (35).

The host-virus relationship between *Polaribacter* ASV_3133 and 13 of group 2 Bacteroidetes viruses in the CIF treatment was the most prominent example of possible host-virus pairs (Fig. 3 and 4; Table 2), as previously unknown but possible roles of viruses for their host metabolism were elucidated. *Polaribacter* ASV_3133 grew specifically in the microcosm, including in the CIF (Table 1). This is consistent with a previous study that detected *Polaribacter* in natural diatom blooms and their well-known function of utilizing phytoplankton-derived polysaccharides, such as laminarin included in diatom cells, as growth substrates (14, 15, 36). Importantly, the *Polaribacter* ASV was the only Bacteroidetes detected as CIF specific and the 13 of group 2 Bacteroidetes viruses were also CIF specific (Fig. 4; Table 2). Co-occurrence between this ASV and 11 of group 2 Bacteroidetes viruses detected in Osaka Bay samples (Fig. 5A; Table S10) further emphasizes the confidence of these host-virus pairs. Three of these Bacteroidetes vOTUs possessed possible laminarinase (GH16) as the AMG. Considering that diatom cells contain laminarin as the major carbon storage material (51), these viruses are presumed to obtain the energy required for proliferation by promoting the utilization of sugars generated by laminarin degradation by the host. In addition to the other vOTUs, previously isolated *Polaribacter* viruses derived from a diatom bloom sample (29, 48) lack genes for GH16, suggesting diverse propagation strategies in viruses infecting Bacteroidetes hosts responding to diatom blooms.

Rhodobacteriales (Alphaproteobacteria) bacteria, *Nereida ignava*, and *Planktomarina temperata* are ecologically important because they are abundant, potentially bloom-responding taxa in coastal areas (10, 38, 40). Although neither *Nereida* nor *Planktomarina* viruses have been isolated (as of October 2022 in Virus-Host DB) (https://www.genome.jp/virushostdb/), we identified one potential virus that might be capable of infecting either or both of them. *N. ignava* and *P. temperata* were the only Rhodobacteriales species that were identified as CIF specific, and vOTU H1_1_NODE_12 was predicted to be the CIF-specific Rhodobacterial virus (Table 2). In particular, the co-occurrence between the *P. temperata* ASV and the vOTU in Osaka Bay samples further supports the *P. temperata*-H1_1_NODE_12 pair (Fig. 5B; Table S10). These biological interactions among prokaryotes and prokaryotic viruses possibly occur in Osaka Bay in which *H. akashiwo* forms blooms (34).

Our experiment might also capture potential host-virus pairs unknown by previous culture-dependent viral isolation and even by genomics-based *in silico* prediction. The only Bacteroidetes bacterial ASV_2651 detected as HIF specific is of the NS9 marine group (Fig. 3; Table 1), which is an uncultured bacterial clade of Bacteroidetes but is abundant in coastal areas (43, 52). HIF-specific Bacteroidetes vOTU (mtsOBV_16Mar_NODE_41) co-occurred with the ASV (Fig. 4; Table S6) in the microcosm samples, suggesting that they are potential a host-virus pair.

Although Alteromonadales (Gammaproteobacteria) ASVs were detected as CIF-specific and HIF-specific ASVs (Fig. 3), no CIF-specific or HIF-specific vOTUs were assigned as Alteromonadales viruses (Fig. 4), possibly due to insufficient viral isolates infecting them and the limitations of current host prediction methods. Indeed, viruses infecting *Glaciecola amylolytica* (CIF specific) and *Psychrobium* (HIF specific) have not been isolated (as of October 2022 in Virus-Host DB) (https://www.genome.jp/virushostdb/). However, host-unknown vOTUs (Original_NODE_59 and mtsOBV_16Mar_NODE_383) in HIF treatments were correlated with the HIF-specific Alteromonadales ASVs in both the microcosm and natural seawater samples (Fig. 3 and 4; Fig. S8; Tables S10 and S11), indicating the possibility that the host-unknown HIF-specific vOTUs include Alteromonadales viruses. Such a correlation is less confident in predicting host-virus relationships compared to genome-based prediction methods (53).

Collectively, based on the difference in predicted host-virus pairs between the treatments, we concluded that globally distributed bloom-forming phytoplanktonic species, *H. akashiwo* and *Chaetoceros*, promote distinct prokaryotes and associated viruses. As genomes of some of the *Chaetoceros*-related viruses carry AMGs for glycoside hydrolase family such as GH16, it can be postulated that those prokaryotic viruses stimulate polysaccharide utilization of host prokaryotes prior to the host lysis. Conversely, AMGs were not detected in the genome of *H. akashiwo*-related counterparts. Although those viruses were not involved in polysaccharide degradation, similar to other typical prokaryotic viruses, they can suppress host transcription and promote nucleic acid synthesis (27), resulting in changes in endo-metabolites eluted from lysed cells. Therefore, we propose that the process of the DOM composition changes following bloom demise would vary among bloom-forming species through the presence or absence of particular prokaryotic viruses. If so, due to the different DOM composition in surrounding environments, subsequently increased prokaryotes and their viruses would also vary, resulting in further alteration of biogeochemical cycling during blooms. Since our focal phytoplankton, *H. akashiwo* and *Chaetoceros,* were reported to emerge simultaneously or successively in the same coastal area (5), the prokaryotic and viral dynamics observed in this study would further help to understand coastal microbial ecosystems. The potential alterations in biogeochemical cycling through changes in prokaryotic and viral dynamics should be elucidated in future research.

## MATERIALS AND METHODS

### Culturing of phytoplankton and collection of intracellular fractions

*H. akashiwo* strain NIES-293 and *Chaetoceros* sp. strain NIES-3717 were purchased from the National Institute for Environmental Studies (NIES, Tsukuba, Japan) and grown axenically in 300 mL of f/2 medium (54) at 20°C under 10/14-h light/dark photocycle conditions at 40 µmol photons m^−2^ s^−1^.

In the coastal ocean, grazers are one of the essential drivers of phytoplankton bloom decline (4, 55). The resulting cell breakage causes the elution of intracellular fractions derived from phytoplankton (56). To obtain intracellular fractions, we physically broke phytoplanktonic cells according to the method of a previous study (12). Phytoplanktonic cells in the exponential growth phase were collected by centrifugation at 420 × *g* for 10 min (High Capacity Bench-top Centrifuge LC-220, TOMY SEIKO, Tokyo, Japan). The cell pellet was washed twice with autoclaved aged seawater and resuspended in 30 mL of the same. The washed cells were fractured using a bead-beater (µT-12, TAITEC, Saitama, Japan) (3,200 rpm, 60 s × 2) with 1 cm^3^ of glass beads (0.5 mm diameter, AS ONE, Osaka, Japan), which had been pre-combusted at 200°C for 2 h. Cell breakage of the two phytoplanktonic species was confirmed using microscopy. Crushed cellular debris including membranous fractions and glass beads were removed by centrifugation (13,000 × *g* for 15 min), and contaminated glass beads in the supernatant were subsequently removed by filtration using a polyvinylidene cellulose acetate filter (25 mm diameter, 0.2 µm pore size, Millipore, Billerica, MA, USA) to obtain 30 mL of dissolved intracellular fractions including organic matter. The dissolved intracellular fractions derived from *Chaetoceros* sp. and *H. akashiwo* were named CIF and HIF, respectively. CIF and HIF were stored at −80°C until use. To measure the carbon concentration in the CIF and HIF, 1.5 mL of each sample was diluted with 10 times the volume of Milli-Q water and pre-filtered with 25 mm diameter, 0.2 µm pore size filters (Millipore). The total organic carbon concentration of these samples was analyzed using a total organic carbon analyzer (TOC-L, Shimadzu, Kyoto, Japan).

### Experimental set up

Approximately 12 L of seawater was collected at 3.8 m depth in Osaka Bay, Japan (34°19′28″ N, 135°7′15″ E), on 31 January 2019. The seawater was pre-filtered through polycarbonate membrane filters (142 mm diameter, 3.0 µm pore size; Millipore) to remove eukaryotic cells. For the microcosm experiments, 1.2 L of the pre-filtered seawater was ﬁltered through polycarbonate membrane filters (142 mm diameter, 0.2 µm pore size; Millipore) to obtain prokaryotic cells and remove the floating (not infecting) viral particles. The prokaryotes and viruses on the filters were re-suspended in 1.2 L of autoclaved seawater in 2 L flasks (nine flasks in total). Residual organic matter on the surface of the flasks was removed by washing with 6 N HCl followed by Milli-Q water before use. Five milliliters of CIF or 4.43 mL of HIF was added to the three flasks containing the prokaryotic fraction, respectively. The input carbon concentration (84 µmol C/L final concentration) was adjusted to match the concentration caused by natural phytoplankton blooms (57). For preparing control flasks, no additional organic matter was added to the rest of the three flasks. Control, CIF-treated, and HIF-treated flasks were thereafter referred to as control, CIF treatment, and HIF treatment, respectively. Triplicate flasks of each treatment were designated as replicate flasks I–III. Nine flasks in total were incubated at 20°C under a 14.5/9.5-h light/dark photocycle condition at 150 µmol photons m^−2^ s^−1^ for 7 days. Temperature and light/dark conditions were consistent with the representative environmental conditions of the bloom season in Osaka Bay, Japan.

### Counting of prokaryotic cells and viral particles

Incubated control, CIF-treatment, and HIF-treatment flasks were homogenized by mixing once a day before subsampling. For counting prokaryotic cells and viral particles, 1,920 µL of samples was taken from each flask every day and added to glutaraldehyde (1% final concentration) for fixation at 4°C. The fixed prokaryotic cells in half of each fixed sample were stained with SYBR Green I (final concentration 1×; Thermo Fisher Science, Waltham, MA, USA) for 20 min at room temperature under dark conditions. For viral counting, prokaryotic cells in the remaining aliquots of fixed samples were removed by filtration with a cellulose acetate filter (13 mm diameter, 0.2 µm pore size; Advantec Toyo Kaisha, Ltd., Tokyo, Japan). The filtrates were subjected to staining with SYBR Green I (final concentration 1×) for 20 min at 80°C, followed by incubation at room temperature according to a previous study (58). At least 10,000 stained cells and viral particles were counted using an S3e Cell Sorter (Bio-Rad, Hercules, CA, USA) and analyzed using FlowJo (Becton, Dickinson and Company, Franklin Lakes, NJ, USA), according to the manufacturer’s instructions. The CIF-treatment flask replicate III sample on day 3 was lost owing to a technical error. Mann–Whitney *U* tests were performed to compare the cell and viral abundance of samples in the same phase (early, middle, and late) among the control, CIF-treatment, and HIF-treatment flasks.

### DNA extraction and sequencing

For prokaryotic community structure analysis, 1,800 µL of the sample was subsampled from each flask every day (72 samples in total), and the cells were collected on polycarbonate membrane filters (13 mm diameter, 0.2 µm pore size; Advantec Toyo Kaisha, Ltd.) and stored at −30°C until DNA extraction. For community composition in original seawater, prokaryotic cells in 100 mL of seawater were collected on polycarbonate filters (47 mm diameter, 0.2 µm pore size; Advantec Toyo Kaisha, Ltd.), and the filters were stored at −30°C until DNA extraction. DNA was extracted using previously published methods (35). The 16S rRNA genes were amplified using a primer set for the V3–V4 hypervariable region of prokaryotic 16S rRNA genes (59) with added overhang adapter sequences at each 5ʹ-end, according to the manufacturer’s sample preparation guide (https://support.illumina.com/documents/documentation/chemistry_documentation/16s/16s-metagenomic-library-prep-guide-15044223-b.pdf). The amplicons were sequenced using the MiSeq Reagent Kit, version 3 (2 × 300 bp read length; Illumina, San Diego, CA, USA).

For viral community structure analysis, 100 mL of the sample was subsampled from each flask on days 0, 1, 3, 5, and 7 (45 samples in total). To analyze the viral composition in the original seawater, 100 mL of 3.0 µm pre-filtered seawater was stored. Prokaryotic cells were removed from the 46 samples by filtration with 0.22 µm pore Sterivex filtration units (SVGV010RS, Millipore), and filtrates were stored as viral fractions at 4°C until DNA extraction. The viruses in the filtrate were concentrated via FeCl_3_ precipitation and purified by CsCl density centrifugation, followed by DNase treatment as described in a previous study (60). DNA extraction was performed using the combination of xanthogenate-SDS and PCI/CIA protocols described in a previous study (61). Extracted DNA was stored at −30°C. Libraries were prepared using a Nextera XT DNA sample preparation kit (Illumina) according to the manufacturer’s protocol, except for the amount of used DNA; we used 0.25 ng of viral DNA as input, while the manufacturer’s recommendation was 1 ng of DNA. To obtain reference viral genomes in the microcosm experiments, we sequenced reads from one flask in which we observed the most abundant viral particles for each treatment. The remaining samples (two flasks per treatment) were sequenced for mapping onto the reference genomes to analyze the dynamics of viruses. Libraries for two samples (control replicate II on days 1 and 3) could not be prepared; thus, we removed them for the following procedure. The retained libraries were sequenced using MiSeq V3 (2 × 300 bp) (Illumina).

### Sequence processing and ASV generation

From the original seawater sample, 25,220 reads were obtained (Table S1). During the microcosm experiments, 345,382 reads in the control and 308,474 and 683,932 in the CIF- and HIF-treatment flasks, respectively, were obtained on average per flask (Table S1). The DADA2 plugin (62) of QIIME2 was used for quality filtering, denoising, pair-end merging, and construction of a feature table of ASVs. Taxonomic assignment of ASVs was performed using the SILVA (release 138) reference database (63) and those assigned to mitochondrial or chloroplast sequences were removed from the feature table for further analyses. The singleton ASVs were removed during this step, and the control flask replicate I sample on day 1 was removed from further analyses as the obtained reads were less than 5,000.

### Analysis of community structure and dynamics of prokaryote ASVs

Rarefaction curves were constructed for all samples using the “iNEXT” package in R (64). Sequence depths were likely sufficient for all samples as the ASVs in those samples were almost saturated by 10,000 reads (Fig. S2); thus, we retained all the samples, except for control flask replicate I on day 1 (see above) for further analyses. For ASV composition, all sequence data were rarefied to the lowest sample depth of 9,450 reads per sample, the Bray–Curtis dissimilarity index was calculated using “vegan” in R for pairwise comparisons of the prokaryotic communities in all samples, and then visualized by PCoA using the “stats” package in R. ANOSIM was performed with the Bray–Curtis dissimilarity scores to assess the significance of the differences between early (days 0–1) and middle-late (days 2–7) samples and that among middle-late samples between the control, CIF, and HIF treatments using the R package “vegan.” *P*-values for multiple comparisons were corrected using the Bonferroni method.

The relative abundance of each ASV was calculated by dividing the read count of the ASV by the total read count of each sample. The approximate cell number of each ASV was obtained by multiplying the relative abundance (0–1) by the total number of prokaryotic cells. Certain ASVs were regarded as abundant if they passed both of the following criteria: (i) they ranked in the top 20 in the approximate cell number at least 1 day after day 1 in all triplicate flasks and (ii) their approximate cell number became more than twice as high as day 0 at least once. We used LEfSe (65) to test whether the differences in the approximate cell number of ASVs of interest among treatments were statistically significant, using treatment as a class and triplicate flasks as subclasses.

### Survey of prokaryote ASVs abundance in amplicon data set from Osaka Bay natural seawater samples

To confirm that ASVs of interest were detected in the natural environment, we analyzed their abundance at the sampling site in this study, Osaka Bay. Raw amplicon reads of 16S rRNA gene sequences obtained from natural seawater samples collected monthly in Osaka Bay between May 2015 and November 2016 (25) were retrieved from the DNA Data Bank of Japan (DDBJ) under project number PRJDB10879. These reads were quality-trimmed and merged, and chimeras were removed following the pipeline described in a previous study (35). Quality-controlled reads were mapped on ASVs of our interest with 100% identity using VSEARCH (66).

### Sequence processing, vOTU clustering, and taxonomic classification

We obtained 17,171,182–25,143,457 reads and 1,159,097–3,806,753 viral metagenomic reads from deeply sequenced and less deeply sequenced samples, respectively (Table S2). Three samples (control replicate III on day 1 and HIF-treatment replicate II on days 1 and 3) were removed from further analysis because of their low number of reads (<10,000 reads). Without quality filtering, all metagenomic reads of each sample (43 samples in total) were individually assembled and scaffolded using SPAdes (67) with default k-mer lengths. Scaffolds longer than 1 kb were used for subsequent analyses. Among these, scaffolds with lengths between 1 and 10 kb were used only for abundance estimation. Scaffolds longer than 10 kb were considered partial or almost complete genomes and then used for host prediction, abundance estimation, and functional characterization.

Prokaryotic scaffolds contaminated in the viral fractions were detected using VirSorter2 and then removed (68). To remove redundancy, these virus-derived scaffolds were clustered into vOTUs based on the average nucleotide identity (ANI) (>95%) using cd-hit (69). The quality of the vOTUs was assessed using CheckV (70) with default settings. Although low-quality vOTUs are likely to be split fragments of viral genomes, leading to overestimation of the number of vOTUs, these were also retained together with high- and middle-quality scaffolds, and we did not use them to compare the number of vOTUs among treatments. The vOTUs judged as “host” regions by CheckV were removed in this step. Consequently, 184 vOTUs generated from the original seawater sample and 296 vOTUs from the microcosm samples were retained (Tables S3 and S4). Detection of the lysogenic virus was also performed using CheckV. For taxonomic classification of the vOTUs, they were assigned to gOTUs by ViPTree, as described previously (25, 45, 71).

### Host prediction of vOTUs obtained in this study

The host prediction of these vOTUs was performed following a previously described method (25, 46, 53). Briefly, putative host groups were assigned based on their similarity to the viral genomic sequence set collected in previous studies (25, 45). If a vOTU was classified into the gOTU, which included host-known viruses, the host group was assigned to the vOTU. Additionally, for vOTUs that were not classified into any host-known gOTUs, host prediction was performed based on nucleotide sequence similarity, CRISPR-spacer matching, and tRNA matching with prokaryotic genomes, as described previously (25, 46).

### Read mapping for abundance estimation of vOTUs

To estimate the relative abundance of each virus, we performed read-mapping analysis. To improve the annotation of each read, mapping accuracy, and mapping rate, we used all 79,391 scaffolds that were longer than 1 kb obtained in this study and 10,759 previously published viral genomes [8,948 environmental viral genomes obtained from previous studies (25, 45) and 1,811 reference viral genomes (45) registered in the NCBI RefSeq database] as reference sequences. Although some of the 79,391 scaffolds were classified into host or viral sequences by VirSorter2 and CheckV, as described above, all the scaffolds including host regions were retained in this step to improve the accuracy of read annotation and read mapping. The reference sequences were clustered into 56,872 non-redundant genomes based on ANI (>95%) using cd-hit. Before mapping, the quality-control procedure of raw reads was performed using fastp (72) (reads were scanned with four base pairs of the sliding window from the 5′ end and the residual base pairs were dropped if the mean quality score was below 20). These quality-controlled reads were mapped onto the 56,872 scaffolds using Bowtie2 (73) with a parameter “--score-min L,0,–0.3.” Fragments per kilobase per mapped million reads (FPKM) were calculated using CountMappedReads2 (https://github.com/yosuken/CountMappedReads2). We regarded the reads mapped on scaffolds or genomes, which were judged as “viral” by both VirSorter2 and CheckV as virus-derived reads, and the reads mapped on the rest of scaffolds as host-derived reads. The reads recruited on neither the host nor the viral scaffold could be regarded as potential virus-derived reads that were not used for assembly.

### Analysis of vOTU dynamics

To analyze the dynamics of the absolute viral density, we estimated the approximate particle number, as described in the prokaryotic community analysis. First, we clustered >10 kb viral scaffolds, including vOTUs obtained in this study and the published viral genomes, which were judged as “viral” by both VirSorter2 and CheckV with the 95% identity of ANI again. We then applied the formula shown below to the resultant 9,413 vOTUs. (i) We calculated the relative abundance of each of the 9,413 vOTUs. The FPKM value of each vOTU was divided by the total FPKM value of >1 kb of the viral scaffolds. (ii) FPKM-based relative abundance was calibrated using the number of potential virus-derived reads. (iii) The calibrated FPKM-based relative abundance was multiplied by the total abundance of viral particles.


$$P\left(i\right)=\frac{FPKM\left(i\right)}{FPKM\left(viral\right)}\times \frac{R\left(viral\right)}{R\left(viral\right)+R\left(potential\;v\right)}\times P\left(viral\right)$$

where *P(i*) is the approximate particle number of vOTUi. FPKM*(i)* is the FPKM value of vOTUi. FPKM(viral) is the total FPKM value of >1 kb viral scaffolds. *R*(viral) is the number of reads mapped on >1 kb viral scaffolds. *R*(potential *v*) is the number of potential virus-derived reads (reads were not recruited on either host or viral scaffolds). *P*(viral) is the total abundance of viral particles.

Next, we performed a statistical analysis based on the approximate particle number to detect vOTUs that increased during the microcosm experiments. The approximate particle number of each vOTU in the day 0 samples was compared with that after day 1 by LEfSe (day 0 and after day 1 were set as class, and the triplicate flasks were set as subclass). vOTUs that showed a significantly higher density after day 1 were retained. Previous studies have reported that the average burst size of marine bacterial viruses is 50–185 particles per host cell (74, 75). Thus, an approximate value of their average, 100 particles, was used as the minimum threshold to determine whether a vOTU increased by host lysis. The vOTUs whose approximate particle number did not exceed 100 particles/mL on any day were discarded.

### Gene prediction and annotation of vOTUs

The prediction of open reading frames (ORFs) present in the vOTUs using GeneMarkS (76) and homology search of detected ORFs against the NCBI/nr database using GHOSTX (77) were performed using the ViPtree web server (https://www.genome.jp/viptree/). The prediction of AMGs in significantly increased vOTUs was performed using DRAMv (78) with default settings.

### Survey of vOTU abundance in virome data from Osaka Bay natural seawater samples

To confirm whether these retained vOTUs exist in the environment, their abundance in samples collected monthly from May 2015 to November 2016 in Osaka Bay (25) was analyzed by read mapping; a vOTU was regarded as present if even one read was mapped onto it. Raw metagenomic reads were obtained from the DDBJ under the project number PRJDB10879 (25). Quality control of the reads was performed as described above, and the metagenomic reads were mapped to the sequence of vOTUs of interest with 95% identity using Bowtie2 with a parameter “--score-min L,0,–0.3.” The FPKM values were calculated as described above.

### Co-occurrence network analysis between ASVs and vOTUs in Osaka Bay natural seawater samples

Spearman correlations between ASVs and vOTUs of interest were calculated based on their abundance in Osaka Bay samples using the local similarity analysis program (79). Only samples that covered both prokaryotic and viral data sets were used for this analysis (17 samples), and time delay was not permitted. ASV-vOTU pairs that met *r* > 0.6 (*P* < 0.01, *q* < 0.05) were regarded as having a significant positive correlation.

## Data Availability

The sample information obtained in this study was deposited in the DNA Data Bank of Japan (DDBJ) under project number PRJDB14359. Raw sequence reads can be found under accession number DRA014887.

## SUPPLEMENTAL MATERIAL

Supplemental figures msystems.00949-23-s0001.pdf Figures S1-S8.

Supplemental tables msystems.00949-23-s0002.xlsx Tables S1-S12.

ASM does not own the copyrights to Supplemental Material that may be linked to, or accessed through, an article. The authors have granted ASM a non-exclusive, world-wide license to publish the Supplemental Material files. Please contact the corresponding author directly for reuse.
